# NADPH Oxidase 4 Deficiency Reduces Aquaporin-2 mRNA Expression in Cultured Renal Collecting Duct Principal Cells via Increased PDE3 and PDE4 Activity

**DOI:** 10.1371/journal.pone.0087239

**Published:** 2014-01-23

**Authors:** Eric Féraille, Eva Dizin, Isabelle Roth, Jean-Paul Derouette, Ildiko Szanto, Pierre-Yves Martin, Sophie de Seigneux, Udo Hasler

**Affiliations:** 1 Departments of Cellular Physiology and Metabolism, University Medical Center, Geneva, Switzerland; 2 Service of Nephrology, Department of Medical Specialties, University of Geneva, Geneva, Switzerland; 3 Service of Endocrinology, Diabetology, Hypertension and Nutrition, Department of Medical Specialties, University of Geneva, Geneva, Switzerland; University College London, United Kingdom

## Abstract

The final control of renal water reabsorption occurs in the collecting duct (CD) and relies on regulated expression of aquaporin-2 (AQP2) in principal CD cells. AQP2 transcription is primarily induced by type 2 vasopressin receptor (V_2_R)-cAMP-protein kinase A (PKA) signaling but also by other factors, including TonEBP and NF-κB. NAPDH oxidase 4 (NOX4) represents a major source of reactive oxygen species (ROS) in the kidney. Because NOX-derived ROS may alter PKA, TonEBP and NF-κB activity, we examined the effects of NOX4 depletion on AQP2 expression. Depleted NOX4 expression by siRNA (siNOX4) in mpkCCD_cl4_ cells attenuated increased AQP2 mRNA expression by arginine vasopressin (AVP) but not by hypertonicity, which induces both TonEBP and NF-κB activity. AVP-induced AQP2 expression was similarly decreased by the flavoprotein inhibitor diphenyleneiodonium. siNOX4 altered neither TonEBP nor NF-κB activity but attenuated AVP-inducible cellular cAMP concentration, PKA activity and CREB phosphorylation as well as AQP2 mRNA expression induced by forskolin, a potent activator of adenylate cyclase. The repressive effect of siNOX4 on AVP-induced AQP2 mRNA expression was abolished by the non-selective phosphodiesterase (PDE) inhibitor 3-isobutyl-1-methylxanthine (IBMX) and was significantly decreased by selective PDE antagonists cilostamide and rolipram, but not vinpocetine, which respectively target PDE3, PDE4 and PDE1. Thus, by inhibiting PDE3 and PDE4 activity NOX4-derived ROS may contribute to V_2_R-cAMP-PKA signaling and enhance *AQP2* transcription.

## Introduction

Despite variations of water intake and loss, whole body water homeostasis is maintained within a narrow range by the continuous adjustment of water reabsorption by the kidney collecting duct (CD) [1]. This process critically relies on the kidney's ability to modulate both the corticomedullary osmotic gradient and aquaporin-2 (AQP2) water channel abundance that respectively provide the driving force and the permeability for water reabsorption [2], [3]. Osmotically driven diffusion of water across CD principal cells is dramatically increased by insertion of AQP2 in the apical membrane [1]. Water exits cells via basolateral AQP3 and AQP4 to be returned to the circulatory system [4], [5]. The clinical importance of AQP2 for water reabsorption is illustrated by imbalances of body fluid homeostasis that arise from deregulated AQP2 expression and mutations in the *AQP2* gene [3], [6]. Such dysfunction also highlights the importance of factors that modulate AQP2 expression. The antidiuretic hormone arginine vasopressin plays a key role by increasing both *AQP2* transcription as well as AQP2 expression at the apical cell surface [7]. Vasopressin exerts its effects by binding to basolateral G_s_-coupled type 2 vasopressin receptor (V_2_R), eliciting the liberation of G protein α_s_-subunits, activation of adenylyl cyclase (AC) type III and VI and increase of adenosine 3′, 5′-monophosphate (cAMP) concentration and kinase activity, including protein kinase A (PKA) [7], [8]. The effect of vasopressin on *AQP2* transcription is complex and likely relies on the functional interplay between numerous factors [3], [6], [7]. In addition to vasopressin, experimental evidence indicates that several other stimuli affect *AQP2* transcription, including environmental tonicity, insulin, aldosterone and extracellular calcium [2], [3]. These either directly affect the V_2_R-cAMP-PKA pathway or act independently of it.

NAPDH oxidases (NOXs) are major sources of reactive oxygen species (ROS) and are the only enzyme family known to produce ROS as their primary function [9], [10]. To date, five NOX isoforms (NOX1, NOX2, NOX3, NOX4 and NOX5) and two related enzymes (DUOX1 and DUOX2) have been identified. NOX1, NOX2 and NOX4 are expressed in both mouse and human kidney, whereas NOX5 is only expressed in human kidney [11]. Experimental data indicate that NOX4 is the most abundant NOX isoform in the kidney, while NOX1 and NOX2 are expressed at low levels [12], [13]. NOX4 expression is especially high in the tubular cell compartment, predominantly in proximal tubular cells, where it significantly contributes to tubular H_2_O_2_ production [11]. Contrary to other NOX isoforms, NOX4 activity is primarily determined by its abundance [14].

In addition to their bactericidal activities in phagocytic cells, NOX play numerous physiological roles in nonphagocytic cells [15]–[18]_ENREF_8. Interestingly, the activities of several factors that influence AQP2 abundance are also modulated by ROS. Notably, NOX2 and NOX4 have been shown to modulate cAMP-PKA signaling in pancreatic β-cells [19] and endothelial cells [20]_ENREF_13, respectively, indicating that these NOX isoforms may influence the transcriptional regulation of PKA-sensitive gene products. NF-κB, which contains redox-sensitive cysteine residues in its DNA binding domain [21] and whose activity is increased by NOX-derived ROS [22], reduces *AQP2* transcription [23], [24]_ENREF_15. ROS was additionally shown to contribute to activation of tonicity-responsive enhancer binding protein (TonEBP) [25], which may enhance *AQP2* transcription [2], [26]_ENREF_4. All these findings indicate that AQP2 expression may be sensitive to cellular ROS.

The purpose of this study was to investigate the influence of modulated NOX4 activity on renal AQP2 abundance. We proceeded by examining AQP2 expression in NOX4-silenced mpkCCD_cl4_ cells, a mouse cell line that displays essential functionalities characteristic of CD principal cells [3], [27]. NOX4 silencing was found to significantly decrease AQP2 mRNA and protein expression. This effect relied on decreased PKA activity, resulting from increased activities of phosphodiesterases (PDE) 3 and 4 and attenuated cAMP cellular concentration. These findings reveal a novel role for NOX4 in regulating AQP2 abundance.

## Materials and Methods

### Materials

The following antibodies were used: rabbit anti-AQP2 IgG [28] was a kind gift of SØren Nielsen, (Sigma-Aldrich, St. Louis, MI, T9026), rabbit anti-NaKα [29], mouse anti-GAPDH (Millipore, Billerica, MA), goat anti-pCREB (Santa Cruz Biotechnology, Dallas, TX) and rabbit anti-Phospo-PKA substrate (Cell Signaling Technology, Danvers, MA) IgG. Desmopressin, forskolin, IBMX, DPI, H89, rolipram and cilostamide were purchased from Sigma-Aldrich. Zoniporide dihydrochloride was purchased from Tocris Bioscience (Bristol, UK).

### Cell culture and transfection

mpkCCD_cl4_ [27] and mCCD_cl1_ [30] cells were cultured on permeable filters as previously described [30], [31] and transiently transfected using Lipofectamine 2000 (Invitrogen, Carlsbad, CA) with either scrambled (5′-GCCACUCGUUUGUCGCCCUUGUAAA -3′) or *NOX4* siRNA (5′-UUUAGGGACAGCCAAAUGAGCAGGC-3′). Reduced desmopressin (DDAVP, a V_2_R-selective analogue of vasopressin)-inducible expression by siNOX4 was verified using a second siRNA duplex against NOX4 (5′-UUGAGGUUCAGGACAGAUGCAGAUG-3′). All siRNA duplexes were made by Invitrogen. For hypertonic challenge, isosmotic medium (300 mOsmol/kg) was made hypertonic (500 mOsmol/kg) by replacing 150 µl (out of 600 µl) apical medium and 300 µl (out of 1200 µl) basal medium with 1100 mOsmol/kg medium. Medium osmolality was checked using an osmometer.

### RNA isolation and real-time quantitative (Q) PCR

Total RNA from mpkCCD_cl4_ and mCCD_cl1_ cells was isolated using the NucleoSpin RNA II kit (Macherey-Nagel, Düren, Germany). As part of the manufacture's protocol, genomic DNA contamination was eliminated by on-column DNase digestion. Reverse transcription and triplicate Q-PCR amplification reactions were performed as previously described [31]. Primers used are depicted in Table 1. Real-Time PCR amplification efficiency was validated by performing dilution series experiments that yielded linear regression slope values comprised between -3.6 and -3.3. Melting curves revealed a single amplified product for all genes. Q-PCR analysis revealed non-significant variations of ribosomal phosphoprotein P_0_ expression in mpkCCD_cl4_ cells exposed to numerous stimuli [3], validating its use as a reliable internal standard in this cell line. Nevertheless, we compared P_0_ expression levels with those of other commonly used internal standard genes (HPRT1, 18S ribosomal RNA and HSP90α1) in mpkCCD_cl4_ cells transfected or not with siNOX4 and challenged or not with 10^−9^ M vasopressin for 24 h. Threshold (Ct) values for each gene are depicted in Table 2. No significant differences (P>0.05) between experimental groups were observed for any of these genes (Table 3). For this reason, we used P_0_ as an internal standard for all experiments. Data was analyzed as previously described [32]. The fold change of test gene mRNA was expressed as 2^ΔΔCt^, where ΔCt is the difference in threshold cycles for the test gene and P_0_ and where ΔΔCt is the difference of ΔCt between stimulated (and/or cells transfected with siNOX4) and non-stimulated (and/or cells transfected with scramble siRNA) control. All primers were made by Microsynth (Balgach, Switzerland).

**Table 1:** Real-Time PCR primer sequences (mouse).

Targeted gene	Forward	Reverse
P_0_	AATCTCCAGAGGCACCATTG	GTTCAGCATGTTCAGCAGTG
HRPT1	ATGAGCGCAAGTTGAATCTG	CAGATGGCCACAGGACTAGA
18S	CGTCGTAGTTCCGACCATAA	CCCTTCCGTCAATTCCTTTA
HSP90	CCTGGGAACCATTGCTAAGT	GATAGGCCGAGTAGAATCCG
TNFα	GACCCTCACACTCAGATCATCTTCT	CCACTTGGTGGTTTGCTACGA
IκBα	CGGAGGACGGAGACTCGTT	TTCACCTGACCAATGACTTCCA
NOX2	TCAACTACTATAAGGTTTATGATGATGG	CAGATATCTAAATTATGCTCTTCCAAA
NOX4	CCTGCTCATTTGGCTGTCCCTA	CGGCTACATGCACACCTGAGAA
DUOX1	AGAGGGTCATTGCCACCTAC	TACTCCGGAGGAGTTTGCTT
DUOX2	TTCTCTGGCTGACAAGGATG	AACATCAGGCGGGACTTATC
AQP2	CTTCCTTCGAGCTGCCTTC	CATTGTTGTGGAGAGCATTGAC
AR	AGTGCGCATTGCTGAGAACTT	GTAGCTGAGTAGAGTGGCCATGTC
BGT1	CTGGGAGAGACGGGTTTTGGGTATTACATC	GGACCCCAGGTCGTGGAT
SMIT	CCGGGCGCTCTATGACCTGGG	CAAACAGAGAGGCACCAATCG
NaKα	TCCCTTCAACTCCACCAACAA	TTTGGGCTCAGATGCATTTG
NaKβ	TCGGAGAAGAAGGAGTTTTTGG	GCAGCCATAAAATATCACGTAGAACA
HNF3	ATGAGAGCAACGACTGGAACAG	TGCTGACAGGGACAGAGGAGTA
UT-A1	CTCCTCCTCACAAGCAACAA	TTCACTGCGTCTCACTGTCA
AC6	GACCAAGGACTCTAAGGCATTCC	CACCCCGGTTGTCTTTGCT
V_2_R	CGTGGGATCCAGAAGCTCC	GGCTAGCCAGCAGCATGA

**Table 2:** Comparison of mean threshold cycles (Ct) between cells transfected with either scramble siRNA or siNOX4 and challenged or not (Ctl) with desmopressin (VP).

Gene	scramble siRNA				siNOX4			
	Ctl		VP		Ctl		VP	
	Ct	Ct_stim_/Ct_ct_l	Ct	Ct_stim_/Ct_ct_l	Ct	Ct_stim_/Ct_ct_l	Ct	Ct_stim_/Ct_ct_l
Po	17.62±0.14	1.0	17.52±0.15	0.99±0.01	17.76±0.18	1.01±0	17.61±0.14	1.0±0
HPRT1	21.57±0.12	1.0	21.55±0.11	1.0±0	21.37±0.15	0.99±0.01	21.37±0.13	0.99±0.01
18S	10.71±0.25	1.0	10.52±0.3	0.98±0.01	10.61±0.26	0.99±0.01	10.75±0.13	1.01±0.02
HSP90	17.87±0.16	1.0	17.76±0.21	0.99±0	17.86±0.22	1.0±0	17.93±0.17	1.0±0

Ratios of Ct values obtained in stimulated and control cells (Ct_stim_/Ct_Ctl_) are also shown. Data are from seven independent experiments.

**Table 3:** Comparison of Student's t-test values between stimulated groups [cells transfected with siNOX4 and/or challenged with desmopressin (VP)] and control groups (Ctl) of data depicted in Table 2.

Gene	scramble siRNA				siNOX4			
	Ctl		VP		Ctl		VP	
	Ct	Ct_stim_/Ct_ct_l	Ct	Ct_stim_/Ct_ct_l	Ct	Ct_stim_/Ct_ct_l	Ct	Ct_stim_/Ct_ct_l
Po	-	-	0.62	0.31	0.55	0.11	0.97	0.93
HPRT1	-	-	0.88	0.65	0.32	0.28	0.29	0.15
18S	-	-	0.64	0.21	0.77	0.25	0.89	0.76
HSP90	-	-	0.71	0.23	0.99	0.94	0.79	0.24

### Western blot analysis

mpkCCD_cl4_ cells were homogenized in 1 ml or 100 µl, respectively, of lysis buffer (20 mM Tris-HCl, 2 mM EDTA, 30 mM NaF, 30 mM NaPP, 2 mM Na_3_VO_4_, 0.1% SDS, 1% Triton-X-100, protease inhibitors). Protein concentrations were determined using the bicinchoninic acid protein assay (Thermo Scientific, Pierce, Waltham, MA). Proteins were subjected to SDS-PAGE and blotted onto polyvinylidene difluoride membranes (Immobilon-P, Millipore) using standard methods [29]. Horseradish peroxidase-conjugated secondary antibodies (1∶20'000, Becton Dickinson, Franklin Lakes, NJ) were used for detection of immunoreactive proteins by chemiluminescence (horseradish peroxidase substrate, Immobilon, Millipore). Protein levels were quantified using ImageJ Java-based image processing software after background subtraction. Test protein/GAPDH ratios were calculated and normalized to that of non-stimulated (and/or cells transfected with scramble siRNA) control.

### Cellular cAMP concentration and H_2_O_2_ analysis

For analysis of cAMP concentration, mpkCCD_cl4_ cells were grown to confluence and then incubated in serum- and hormone-free medium prior to 30 min of preincubation in the absence or presence of 200 µM 3-isobutyl-1-methylxanthine (IBMX). Cells were then stimulated or not with DDAVP (10^−9^ M) for an additional 15 min, rinsed with PBS, lysed, and cAMP content was measured using the cAMP Biotrack Enzymeimmunoassay system (GE Healthcare, Little Chalfon, UK) following the manufacturer's instructions, as previously described [33]. For analysis of H_2_O_2_ production, mpkCCD_cl4_ cells grown to confluence on plastic 6 well plates were treated or not with forskolin (5 µM) for 20 h and then treated with diphenyleneiodonium (DPI, 1 mM) for an additional 4 h. Immediately prior to measurements, cells were treated with Amplex Red (Invitrogen, 25 µM) and horse radish peroxidase (Invitrogen, 0.005 U/ml). Amplex red fluorescence assays were followed at 37°C for 90 min in a fluorescence plate reader (λ_ex_ 544 nm, λ_em_ 590 nm) (Fluostar; BMG Labtechnologies). Background counts were subtracted from superoxide counts. Addition of H_2_O_2_ (1 mM) to Amplex Red/horse radish peroxidase mix was used as a positive control and typically yielded values that were 30 fold higher than those obtained in control cells.

### Statistics

Results are given as the mean ± SEM from *n* independent experiments. Each experiment was performed on cells from the same passage and all experiments were performed at least three times. The precise number of experiments performed is indicated in the figure legends. All statistical analyses were performed using Prism software (Graphpad, USA). Significance between two pairs of experiments was determined using a student's t-test.

## Results

### NOX4 deficiency decreases AQP2 expression

**Figure 1:**
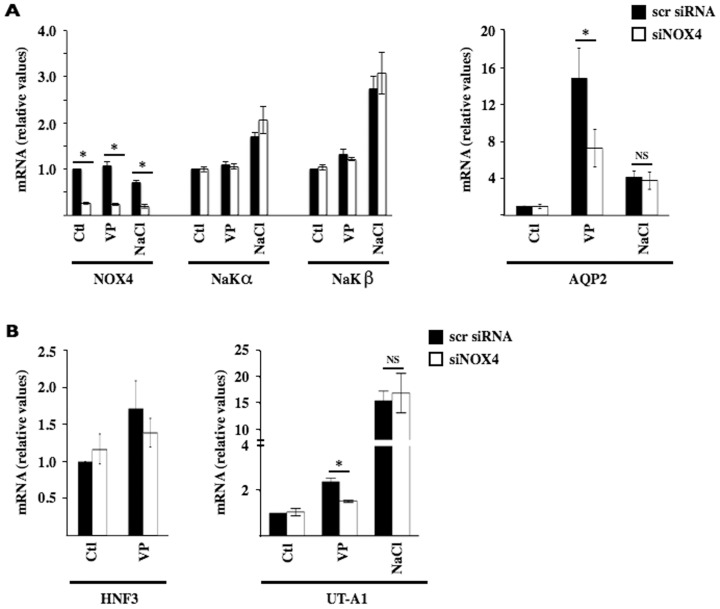
Increased AQP2 expression by a vasopressin analogue is decreased by NOX4 depletion in mpkCCD_cl4_ cells. mRNA expression levels of NOX4, Na,K-ATPase α subunit (NaKα), Na,K-ATPase β subunit (NaKβ) and AQP2 (A) and HNF3 and UT-A1 (B) were compared by Q-PCR in mpkCCD_cl4_ cells transfected with either scramble siRNA (scr) or siRNA against NOX4 (siNOX4) and challenged or not (Ctl) 24 h with either desmopressin, a V_2_R-selective analogue of vasopressin (VP, 10^−9^ M), or NaCl-hypertonic medium (500 mOsmol/kg). Data is represented as fold induction over non-stimulated cells transfected with scramble siRNA and is expressed as the mean ± SEM of six independent experiments. *P≤0.05; NS: no significant differences.

We have previously demonstrated by Amplex Red assay that basal levels of H_2_O_2_ produced in cultured CD principal cells is decreased two-fold by siRNA targeting NOX4 (siNOX4) [34]. We investigated the possibility that the absence of NOX4 might alter intracellular signaling that controls AQP2 expression. We proceeded by comparing the effect of siRNA targeting NOX4 (siNOX4) on AQP2 mRNA expression in mpkCCD_cl4_ cells challenged or not with either desmopressin (DDAVP), a V_2_R-selective analogue of vasopressin that increases endogenous AQP2 mRNA and protein abundance [35], or hypertonicity, which also increases AQP2 expression but to a lesser extent [31] and via different mechanisms [2], [3]. We have previously shown [32] that AQP2 mRNA expression in mpkCCD_cl4_ cells progressively increases in response to DDAVP to reach maximal levels after 8 h of challenge. High mRNA expression levels are maintained for at least 24 h, at which time robust AQP2 protein expression is observed. Maximal stimulation was achieved in cells challenged with 10^−9^ M DDAVP. We have also shown that AQP2 mRNA and protein expression are first decreased after 3 h of hypertonic challenge (≥350 mOsmol/kg) but then increased after 24 h of hypertonic challenge [23], [26], [31]. For this reason, we directly compared the effects of siNOX4 on increased AQP2 expression by either DDAVP (10^−9^ M) or NaCl-hypertonic medium (500 mOsmol/kg) by challenging cells with either stimulus for 24 h. Hypertonicity, but not DDAVP, slightly increased mRNA expression of NaK-ATPase α and β subunits (NaKα and NaKβ), used as comparative controls (Figure 1A). Neither mRNA species was affected by siNOX4. Increased AQP2 mRNA expression induced by DDAVP was significantly decreased by about 50% by siNOX4 while increased expression elicited by hypertonicity was not (Figure 1A). Similar attenuation of the DDAVP, but not hypertonic, response by siNOX4 was observed in mCCD_cl1_ cells (not shown), another renal CD principal cell line that displays major functional characteristics of CD principal cells [30]. We examined the effect of siNOX4 on other vasopressin-responsive genes. Vasopressin was previously shown to increase HNF3 and UT-A1 mRNA expression in CD cells [36]–[38]. DDAVP increased mRNA levels of both genes in mpkCCD_cl4_ cells, although to smaller extents than for AQP2 (Figure 1B). Similar to DDAVP-induced AQP2 expression, this response was decreased by siNOX4 (Figure 1B). UT-A1 mRNA expression may be regulated by osmolality in addition to vasopressin *in vivo* and *in vitro* [39], [40] and its expression was significantly increased by hypertonicity in mpkCCD_cl4_ cells (Figure 1B). Similar to hypertonicity-inducible AQP2 expression, enhanced UT-A1 mRNA expression by hypertonicity was not altered by siNOX4. These results indicate that decreased AQP2 mRNA expression by siNOX4 might arise as a consequence of reduced V_2_R signaling.

**Figure 2:**
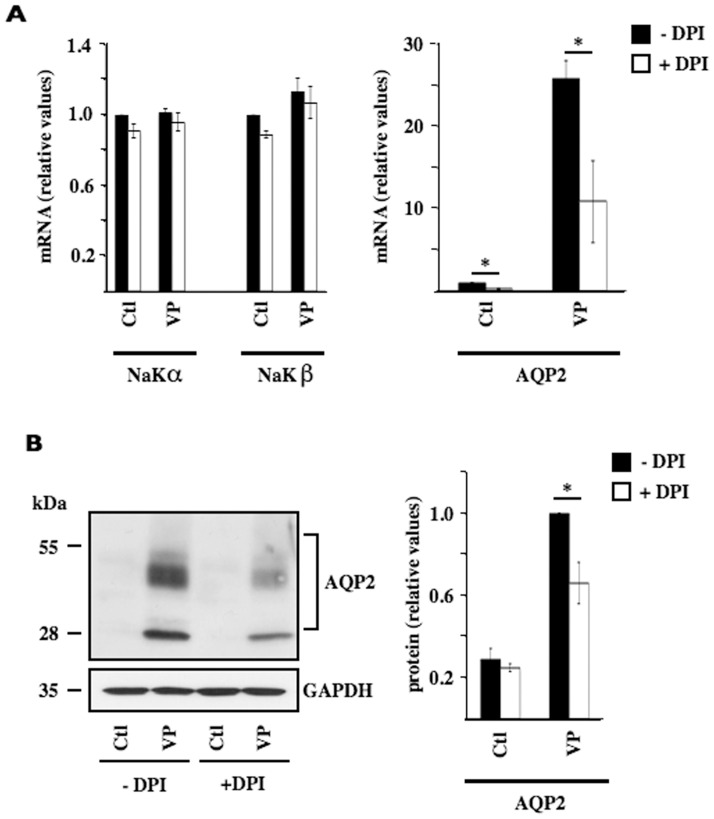
Increased AQP2 expression by a vasopressin analogue is decreased by diphenyleneiodonium. (A) mRNA expression levels of NaKα, NaKβ and AQP2 were compared in mpkCCD_cl4_ cells challenged or not (Ctl) 24 h with desmopressin (VP, 10^−9^ M). Diphenyleneiodonium (DPI, 1 mM) was added or not 30 min prior to VP stimulation. Data is represented as fold induction over non-stimulated cells and is expressed as the mean ± SEM of six independent experiments. (B) AQP2 protein was analyzed by Western blot in cells challenged with VP and DPI as described in (A). GAPDH was used as a loading control. Quantification of AQP2 protein is shown at right. Q-PCR data is represented as fold induction over control cells (i.e cells transfected with scramble siRNA or untreated cells). Western blot data is represented as fold difference of AQP2 signal observed in VP-treated cells in the absence of DPI and is expressed as the mean ± SEM of three independent experiments. Quantification of AQP2 protein was performed on overexposed film (not shown) in order to visualize low levels of control AQP2 protein. *P<0.05.

We next examined whether the antagonistic effect of siNOX4 on DDAVP-inducible AQP2 expression could be reproduced by diphenyleneiodonium (DPI), an inhibitor of flavoproteins, including NOX. Similar to siNOX4, DPI altered neither NaKα nor NaKβ mRNA expression but significantly decreased DDAVP-inducible AQP2 expression by about 50% (Figure 2A). Western blot analysis of AQP2 protein, difficult to achieve in transfected cells, revealed that DPI also decreased DDAVP-inducible AQP2 protein expression (Figure 2B). These results indicate that decreased AQP2 responsiveness to DDAVP by siNOX4 results from decreased ROS production.

### Decreased AQP2 abundance by siNOX4 occurs independently of NF-κB and TonEBP

**Figure 3:**
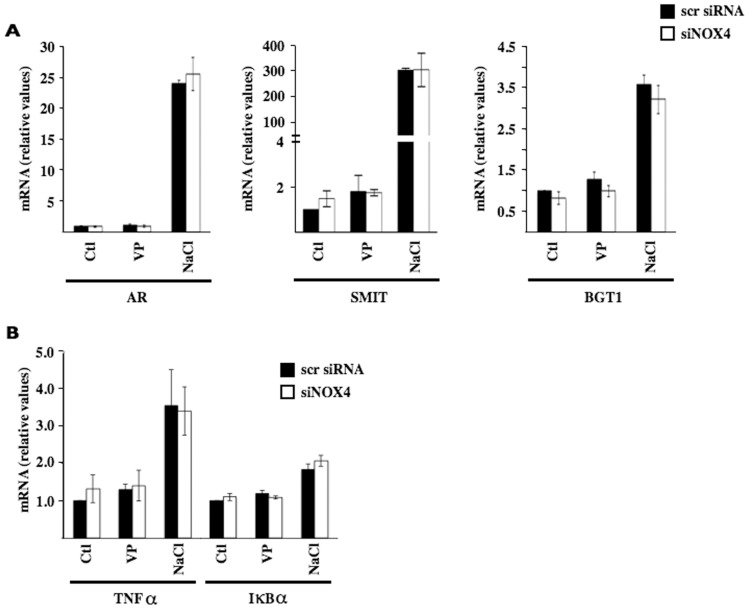
TonEBP and NF-κB activity is not affected by NOX4 depletion. mpkCCD_cl4_ cells transfected with either scramble siRNA or siNOX4 were challenged for 24 h with either desmopressin (VP, 10^−9^ M) or NaCl-hypertonic medium (500 mOsmol/kg). (A) Q-PCR analysis of aldose reductase (AR), sodium-myo-inositol cotransporter (SMIT) and sodium-chloride-betaine cotransporter (BGT1) was performed for investigation of TonEBP activity. (B) Q-PCR analysis of TNFα and IκBα was performed for investigation of NF-κB activity. Data is represented as fold induction over non-stimulated cells transfected with scramble siRNA and is expressed as the mean ± SEM of three independent experiments.

Because ROS has been implicated in TonEBP and NF-κB activation [22], [25], we investigated whether their activities are modulated by siNOX4 in mpkCCD_cl4_ cells (Figure 3). In this cell line, hypertonicity increases both TonEBP and NF-κB activity [23], [26]. In those studies, *AQP2* transcription was shown to be decreased by NF-κB, shortly following challenge, and to be increased by TonEBP after sustained challenge. To exclude the possibility that siNOX4 may decrease DDAVP-induced *AQP2* transcription by attenuating TonEBP activity and/or increasing NF-κB activity, we compared the effects of siNOX4 on mRNA expression levels of TonEBP-dependent genes [aldose reductase (AR), sodium-myo-inositol cotransporter (SMIT) and sodium-chloride-betaine cotransporter (BGT1)] (Figure 3A) and NF-κB-dependent genes (TNFα and IκBα) (Figure 3B) in cells challenged with either DDAVP or hypertonic medium. We have previously shown a good correlation between TonEBP and NF-κB activity and mRNA expression levels of their gene targets in cultured CD principal cells [26], [41]. DDAVP stimulation affected neither TonEBP nor NF-κB activity. In addition, increased activity of these transcription factors by hypertonicity was not affected by siNOX4. Together with the observation that siNOX4 did not affect increased AQP2 mRNA expression by hypertonicity (Figure 1A), these data indicate that repressed AQP2 abundance by siNOX4 in mpkCCD_cl4_ cells does not arise from altered TonEBP or NF-κB activity.

### siNOX4 increases phosphodiesterase 3 and 4 activity and decreases CREB phosphorylation

**Figure 4:**
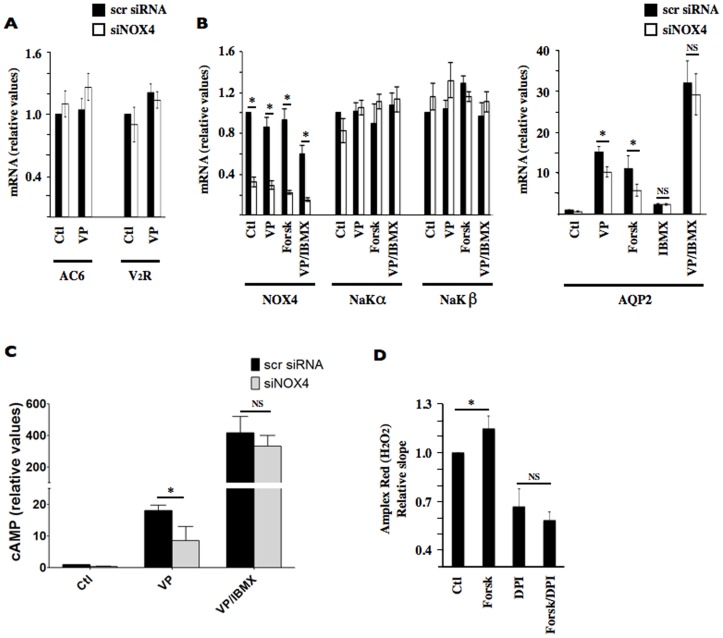
NOX4 depletion decreases cAMP concentration and AQP2 expression in a phosphodiesterase-dependent manner in mpkCCD_cl4_ cells. (A) mRNA expression levels of type 2 vasopressin receptor (V_2_R) and adenylyl cyclase (AC) type VI were compared by Q-PCR in mpkCCD_cl4_ cells transfected with either scramble siRNA or siNOX4 and challenged or not (Ctl) with desmopressin (VP, 10^−9^ M) for 24 h. Data is represented as fold induction over non-stimulated cells transfected with scramble siRNA and is expressed as the mean ± SEM of six independent experiments. (B) mRNA expression levels of NOX4, Na,K-ATPase α subunit (NaKα), Na,K-ATPase β subunit (NaKβ), and AQP2 were compared by Q-PCR in cells transfected with either scramble siRNA or siNOX4 and challenged or not (Ctl) with either VP (10^−9^ M) or forskolin (5 µM) for 24 h, with the nonselective phosphodiesterase inhibitor IBMX (200 µM) for 6 h, or first with VP for 18 h and then with IBMX for an additional 6 h. Data is represented as fold induction over non-stimulated cells transfected with scramble siRNA and is expressed as the mean ± SEM of six independent experiments. (C) cAMP concentration was measured in cells transfected with either scramble siRNA or siNOX4, challenged or not with IBMX for 30 min and then challenged or not with VP for an additional 15 min. Data is represented as fold induction over untreated cells transfected with scramble siRNA. *P<0.05. NS: non-significant difference. Data is represented as fold induction over non-stimulated cells transfected with scramble siRNA and is expressed as the mean ± SEM of three independent experiments. (D) H_2_O_2_ production measured by Amplex Red in cells pretreated or not (Ctl) with forskolin (5 µM) for 20 h and then treated or not with DPI (1 mM) for an additional 4 h. Data is represented as fold induction over non-stimulated cells and is expressed as the mean ± SEM of four independent experiments. *P≤0.05; NS: no significant differences.

We examined cellular mechanisms that might mediate decreased DDAVP-inducible AQP2 abundance by siNOX4. The effects of siNOX4 could result from a deficient response of an initial step of DDAVP signaling (i.e., V_2_R and/or AC). As revealed by Q-PCR, siNOX4 had no effect on mRNA abundance of either V_2_R or AC type VI, the most abundant AC isoform present in mpkCCD_cl4_ cells [42] (Figure 4A), indicating that the effects of siNOX4 may occur independently of altered V_2_R and AC abundance, at least at the mRNA level. The effect of siNOX4 on AQP2 mRNA expression was similar in cells treated with DDAVP or forskolin, which raises cAMP levels by direct AC activation (Figure 4B). On the other hand, decreased DDAVP-induced AQP2 expression by siNOX4 was abolished by 3-isobutyl-1-methylxanthine (IBMX), a nonselective phosphodiesterase (PDE) inhibitor that raises cAMP concentration (Figure 4B). Analysis of cellular cAMP revealed that increased cAMP concentration by DDAVP was decreased by siNOX4 (Figure 4C). However, siNOX4 did not significantly reduce high cAMP levels observed in cells treated with both DDAVP and IBMX (Figure 4C). Together, data of Figures 4A-C indicate that NOX4-derived ROS decrease cAMP degradation by phosphodiesterases rather than directly affecting AC activity itself. We next investigated whether high cAMP levels reciprocally alter ROS production by measuring Amplex Red oxidation by H_2_O_2_ in cells challenged or not with forskolin. As revealed by this assay, H_2_O_2_ production was slightly, but significantly, increased by forskolin challenge (Figure 4D), indicating that increased cAMP concentration by ROS may be amplified by cAMP itself.

**Figure 5:**
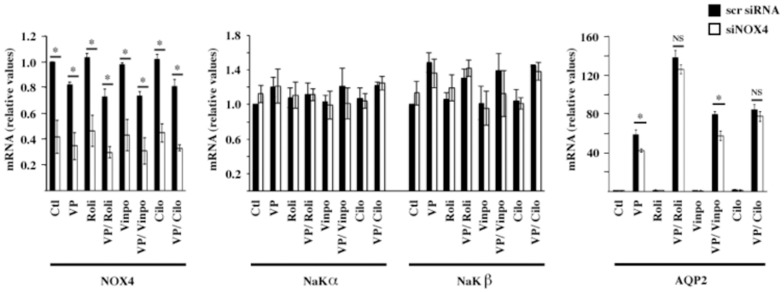
phosphodiesterase-3 and phosphodiesterase-4 mediate decreased AQP2 expression by NOX4 depletion. mRNA expression levels of NOX4, Na,K-ATPase α subunit (NaKα), Na,K-ATPase β subunit (NaKβ) and AQP2 were compared by Q-PCR in mpkCCD_cl4_ cells transfected with either scramble siRNA or siNOX4 and challenged or not (Ctl) with either the selective phosphodiesterase-4 inhibitor rolipram (2 µM), selective phosphodiesterase-1 inhibitor vinpocetine (2 µM) or selective phosphodiesterase-3 inhibitor cilostamide (2 µM) for 30 min prior to an additional 24 h incubation in the absence or presence of desmopressin (VP, 10^−9^ M). Data is represented as fold induction over untreated cells transfected with scramble siRNA and is expressed as the mean ± SEM of three independent experiments. *P≤0.05; NS: no significant differences.

We examined the effects of siNOX4 in the presence of selective cAMP-PDE inhibitors. Analysis of kidney tubule extracts and cultured renal cells indicate that PDE1, PDE3 and PDE4 are the predominant PDE isozymes present in the kidney distal tubule and CD [43], [44]. We examined the effect of siNOX4 on DDAVP-inducible AQP2 expression in the presence of either vinpocetine, cilostamide or rolipram, which selectively inhibit PDE1, PDE3 and PDE4 activity, respectively. While these inhibitors had no effect on NaKα and NaKβ mRNA abundance, increased AQP2 mRNA expression by DDAVP was further increased by all three PDE inhibitors (Figure 5). The extent of decreased DDAVP-induced AQP2 expression by siNOX4 was similar between cells not treated with a PDE inhibitor and cells treated with vinpocetine (Figure 5). The effect of siNOX4, however, was significantly attenuated by addition of either rolipram or cilostamide (Figure 5). This indicates that decreased AQP2 responsiveness to DDAVP by siNOX4 is mediated by increased PDE3 and PDE4 activity.

**Figure 6:**
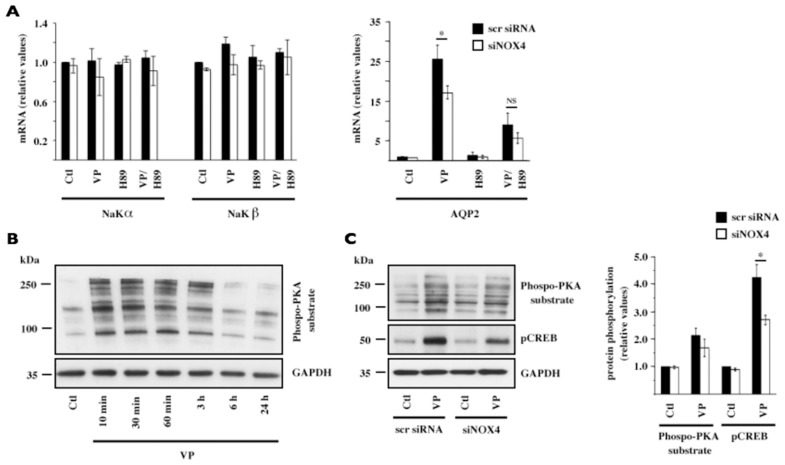
Decreased PKA activity mediates decreased AQP2 expression by NOX4 depletion. (A) mRNA expression levels of NaKα, NaKβ and AQP2 were compared by Q-PCR in cells transfected with either scramble siRNA or siNOX4 and challenged or not (Ctl) with the protein kinase A (PKA) inhibitor H89 (10 µM) for 30 min prior to an additional 24 h incubation in the absence or presence of desmopressin (VP, 10^−9^ M). Data is represented as fold induction over untreated cells transfected with scramble siRNA and is expressed as the mean ± SEM of three independent experiments. (B) left panel: PKA activity in response to VP (10^−9^ M) was examined over time by Western blot analysis of PKA substrate phosphorylation. GAPDH was used as a loading control. A representative blot out of three is shown. (C) PKA substrate phosphorylation and cAMP response binding-protein (CREB) phosphorylation was analyzed by Western blot in cells transfected with either scramble siRNA or siNOX4 and challenged or not (Ctl) with VP (10^−9^ M) for 10 min. GAPDH was used as a loading control. Quantification of proteins is shown at right. Data is represented as fold induction over untreated cells transfected with scramble siRNA and is expressed as the mean ± SEM of three independent experiments. *P≤0.05; NS: no significant differences.

We examined the effect of siNOX4 on PKA activity acting downstream of cAMP. We first compared the effects of the PKA inhibitor H89 on DDAVP-induced AQP2 expression in the absence or presence of siNOX4. In the absence of siNOX4, H89 had no effect on either NaKα or NaKβ mRNA abundance but significantly decreased vasopressin-induced AQP2 mRNA abundance (Figure 6A). While siNOX4 decreased AQP2 mRNA in both the absence and presence of H89, its repressive effect was attenuated by H89 (Figure 6A). We then examined the effect of siNOX4 on PKA activity itself by quantifying phosphorylation of PKA substrates in response to DDAVP. Time course experiments revealed that phosphorylation was greatest after 10 min of DDAVP challenge (Figure 6B). Phosphorylation of PKA substrates occurring at this time was attenuated by siNOX4, although not to a statistically significant extent (Figure 6C). DDAVP-induced phosphorylation of cAMP response binding-protein (CREB), a PKA target, was significantly decreased by siNOX4 (Figure 6C). The importance of a CRE cis element located in the AQP2 promoter was previously demonstrated by its deletion or mutation [45]–[47]. DDAVP was additionally shown to increase CREB nuclear expression in mpkCCD_cl4_ cells [48]. Together, these data indicate that siNOX4 may decrease DDAVP-inducible AQP2 expression by attenuating PKA and CREB activity.

## Discussion

**Figure 7:**
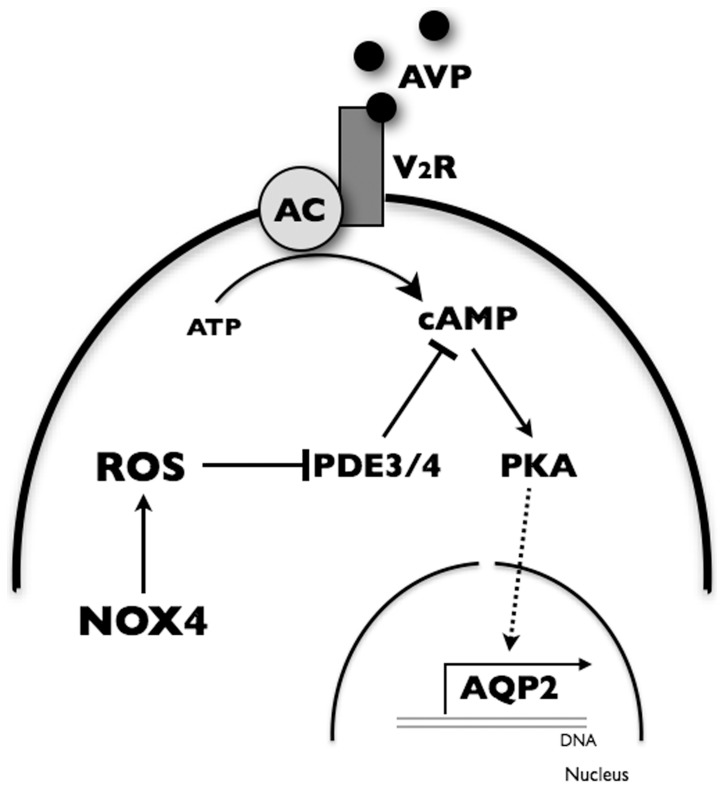
Proposed model of increased AQP2 expression by NOX4-derived ROS in CD principal cells. Stimulation of type 2 vasopressin receptor (V_2_R) by vasopressin raises adenylyl cyclase (AC) activity, cAMP levels and PKA activity, leading to an increase of *AQP2* transcription. NOX4-derived ROS enhances *AQP2* transcription by decreasing cAMP hydrolysis by phosphodiesterase (PDE) type 3 and 4, thereby increasing cellular cAMP concentration and PKA activity.

Although highly expressed in the kidney, the physiological role of renal NOX4, a major source of renal ROS [49], is not understood. Despite its role in H_2_O_2_ production, we have recently shown that its absence is detrimental to tubular cell survival under conditions of cell stress [34]. This view is corroborated by a recent study of the role of NOX4 in murine models of kidney disease [50]. In the present study, we show that AQP2 mRNA abundance is decreased by siNOX4 in mpkCCD_cl4_ cells challenged with DDAVP, which increases cAMP concentration, but not hypertonicity, which increases AQP2 expression independently of a rise in cAMP levels [51], [52]. This repressive effect was found to be associated with increased PDE3 and PDE4 activity and depressed PKA and CREB activity. These findings are summarized in Figure 7 and provide novel evidence that NOX4-derived ROS participate in regulating V_2_R signaling by modulating cAMP concentration.

NOX4 was first identified in the kidney, where it was dubbed ‘Renox’, and has since been found in various other tissues [9], [13]. In the kidney, NOX4 expression is highest in the proximal cortex [11], [50]. Most nonphagocytic cells produce low amounts of ROS that stimulate intracellular signaling pathways [9], [53]. NOX-dependent ROS generation has previously been shown to induce the expression of numerous genes, including TNFα, Ang II and TGF-β1 [9], all of which play important stimulatory functions in the renal CD. Possibly, NOX4 may be expressed in the CD at significantly lower levels than in proximal tubules. These low levels may be necessary to optimize controlled expression of redox-sensitive genes. Interestingly, insulin-induced ENaC activity was prevented by either H_2_O_2_ chelation or NOX inhibition in A6 monolayers derived from amphibian distal nephron [54]. Along with observations of the present study, this raises the intriguing possibility that NOX4 may play a role in whole body fluid homeostasis by modulating both AQP2 and ENaC activity. It is pertinent to mention that ROS acting on CD cells might not necessarily be of autocrine origin alone since H_2_O_2_ diffuses across membranes. The close proximity between CD and PT segments suggests that ROS produced in PT may exit these cells and enter CD cells.

Contrary to NOX1, NOX2 and NOX3, whose enzymatic activities require their association with the transmembrane subunit p22phox and two cytosolic subunits p67phox and p47phox (and additionally p40phox for NOX2), NOX4 associates only with p22phox [18]. NOX4 activity is often assumed to be constitutive and regulated principally at the transcriptional level [14]. However, enhanced activity by p22phox interaction with polymerase delta-interacting protein 2 (Poldip2) [55] and the stimulatory effects of quinone compounds [56] provide new evidence of post-transcriptional regulation. NOX4 abundance itself is most likely regulated, as indicated by inducible NOX4 expression by diverse stimuli, including hypoxia, angiotensin II (Ang II) and TGF-β [9]. Along with data of the present study, this suggests that any stimulus that affects NOX4 activity may transcendingly affect AQP2 expression.

Numerous physiological roles have been attributed to NOX in various tissue systems. NOX2, for instance, was shown to participate in neovascularization, neurotransmitter release and liver fibrosis while NOX1 was reported to play a pivotal role in the pressor response to Ang II and to be involved in inflammatory pain [17], [18]. Physiological roles for NOX4-derived ROS are controversial and may depend on cell type. While NOX4 helps regulate apoptosis and differentiation in some cell types, it regulates cell survival and proliferation in others [18]. Such differences might arise from the influence of ROS on divergent second messenger systems, such as MAP kinase activation or transcription factors, such as NF-κB and AP-1 [9]. NOX-derived ROS have been found to affect NK-κB activity in a variety of physiological contexts in some cell types [22], [57] but not in others [58], [59]_ENREF_55. A previous study has shown that hypertonicity-induced ROS contribute to TonEBP activation in HEK293 cells [25]. In the present study, NOX4 depletion affected neither NF-κB- nor TonEBP-dependent gene expression in mpkCCD_cl4_ cells challenged with hypertonic medium. Pending that altered transcriptional activity may nonetheless have been overlooked, these data indicate that ROS-mediated NF-κB and TonEBP activation may be stimulus and/or cell specific. On the other hand, and importantly, NOX4 depletion reduced vasopressin-inducible cAMP content in mpkCCD_cl4_ cells via increased PDE3 and PDE4 activity. Regulation of cAMP concentration by NOX was previously demonstrated in two different cellular contexts. In one study, enhanced insulin release in response to glucose stimulation in NOX2 KO mice was associated with elevated pancreatic islet cAMP concentration [19]. In another study, performed in cultured umbilical vein endothelial cells, upregulated PDE4A, PDE4B and PDE4D mRNA and cAMP hydrolysis by vasculopathic factors was reduced by siRNA-induced NOX4 silencing [20]. Together, these data indicate that NOX-derived ROS may affect cAMP concentration, and that this effect varies between cell types and/or stimulatory factors. Our analysis further revealed that high levels of cAMP enhance H_2_O_2_ production. This observation made in cultured CD principal cells differs from decreased superoxide generation by raised cAMP levels in isolated neutrophils [60] and pancreatic islets [19]. The reciprocal influence between ROS and cAMP production suggests a link between the redox state of the cell and cAMP levels.

Our study shows that increased AQP2 mRNA expression by DDAVP is decreased by NOX4 deficiency in mpkCCD_cl4_ cells. We did not examine whether siNOX4 similarly affects AQP2 protein expression since, in our hands, analysis of AQP2 protein expression is technically difficult to achieve in mpkCCD_cl4_ cells exposed to Lipofectamine transfection reagent, even in cells displaying high levels of AQP2 mRNA. Because of regulated sequestration of mRNAs that lowers the amount of mRNA available for translation [61], mRNA abundance may not necessarily reflect protein expression levels. On the other hand, AQP2 protein abundance is largely believed to be chiefly regulated by *AQP2* transcription [7]. We have previously found that AQP2 mRNA and protein expression levels are well correlated in mpkCCD_cl4_ cells exposed to increasing concentrations of DDAVP for 24 h [32], i.e. the time point at which AQP2 expression was examined in this study. Moreover, similar to the effects of siNOX4, DDAVP-induced AQP2 protein expression is decreased by the flavoprotein inhibitor diphenyleneiodonium (Figure 2B). Decreased ROS production in NOX4-deficient cells may therefore attenuate vasopressin-induced AQP2 protein expression as a result of its decreased mRNA expression.

While it is well established that *AQP2* transcription is increased by long-term vasopressin challenge in the renal CD [3], the exact mechanism by which this occurs is not obvious. The *Aqp2* gene contains several conserved *cis*-element motifs, including a cAMP-response element (CRE) [7], that bind numerous transcription factors. Deletion and site-specific mutagenesis of the *AQP2* CRE confirmed that this *cis*-element mediates vasopressin-induced *AQP2* transcription [3]. cAMP activates PKA and increased PKA activity by vasopressin may enhance *AQP2* transcription at least partly via activation of a CREB protein [31], [35], [45], [46]. Although one study showed that PKA inhibition had no effect on vasopressin-induced AQP2 protein expression in mpkCCD_cl4_ cells [62], in our hands PKA inhibition reduced both AQP2 mRNA (this study) and protein expression [31] in cells challenged 24 h with DDAVP. This is in good agreement with reduced *AQP2* transcription by PKA inhibition in mpkCCD_cl4_ cells challenged 24 h with DDAVP [63], indicating that PKA helps increase vasopressin-induced *AQP2* transcription, at least in the short-term. Other factors, such as extracellular signal-regulated kinase (ERK) [62] and exchange protein directly activated by cAMP (Epac) [63], have additionally been proposed to mediate *AQP2* transcription by vasopressin. Numerous other putative transcriptional regulators, including CREB proteins, identified by a systems biology-based approach, have recently been proposed to modulate vasopressin-inducible *AQP2* transcription [64]. Together, these studies indicate that increased *AQP2* transcription by vasopressin likely arises as a consequence of the integrative effects of multiple transcriptional regulators.

Results of the present study indicate that in CD principal cells, vasopressin may increase cAMP concentration in two ways: by directly increasing its production by increasing AC activity and by indirectly decreasing its degradation by decreasing PDE activity via ROS. Data of the present study raise the intriguing prospect that NOX4 could represent an attractive target for treatment of disorders associated with deregulation of cAMP signaling in the CCD, such as nephrogenic diabetes insipidus (NDI) and polycystic kidney disease (PKD). Indeed, deficient V_2_R activity associated with X-linked recessive NDI might be circumvented by increasing AQP2 levels via a NOX4-inducible rise of cAMP concentration. Inversely, a reduction of NOX4-mediated cAMP concentration might be beneficial for treatment of PKD, as suggested by the promising effect of strategies that reduce fluid secretion and cell proliferation by lowering cAMP content [65], [66].

In conclusion, we propose that NOX4-mediated production of ROS may enhance V_2_R-cAMP-PKA signaling by attenuating PDE activity. This may increase AQP2 abundance, contributing to the fine-tuning of water reabsorption.
