# Correction: NADPH Oxidase 4 Deficiency Reduces Aquaporin-2 mRNA Expression in Cultured Renal Collecting Duct Principal Cells via Increased PDE3 and PDE4 Activity

**DOI:** 10.1371/journal.pone.0091395

**Published:** 2014-02-27

**Authors:** The PLOS ONE Staff

There are multiple errors in the third paragraph of the Introduction. The third paragraph should read:

"In addition to their bactericidal activities in phagocytic cells, NOX play numerous physiological roles in nonphagocytic cells [15]–[18]. Interestingly, the activities of several factors that influence AQP2 abundance are also modulated by ROS. Notably, NOX2 and NOX4 have been shown to modulate cAMP-PKA signaling in pancreatic β-cells [19] and endothelial cells [20], respectively, indicating that these NOX isoforms may influence the transcriptional regulation of PKA-sensitive gene products. NF-κB, which contains redox-sensitive cysteine residues in its DNA binding domain [21] and whose activity is increased by NOX-derived ROS [22], reduces AQP2 transcription [23], [24]. ROS was additionally shown to contribute to activation of tonicity-responsive enhancer binding protein (TonEBP) [25], which may enhance AQP2 transcription [2], [26]. All these findings indicate that AQP2 expression may be sensitive to cellular ROS."
